# A Case of Bilaterally Inaccessible Subclavian Veins

**DOI:** 10.7759/cureus.73113

**Published:** 2024-11-06

**Authors:** Luka Kiguradze, Jaba Tsivilashvili

**Affiliations:** 1 Internal Medicine, David Tvildiani Medical University, Tbilisi, GEO; 2 Cardiac Intensive Care Unit, Open Heart, Tbilisi, GEO

**Keywords:** icd insertion, implantable cardioverter-defibrillator (icd), subclavian vein occlusion, subclavian vein thrombosis, unilateral subclavian vein cannulation for pacemaker implantation

## Abstract

We present the case of a 69-year-old female with a history of myocardial infarction, ischemic cardiomyopathy, and left bundle branch block, who was scheduled for a cardioverter-defibrillator (CRT-D) implantation. During the procedure, an unexpected left subclavian vein obstruction by a thrombus was encountered, preventing access for lead placement. Further attempts to utilize the right subclavian vein were complicated by the discovery of an acute angle near the brachiocephalic vein, which made lead placement technically unfeasible. Despite multiple attempts to navigate the venous anatomy, the procedure was halted due to the complexity of these combined vascular anomalies. This case illustrates the rare occurrence of both subclavian vein obstruction and an acute venous angle which significantly complicates proper lead placement during CRT-D implantation, emphasizing the importance of preoperative planning and the potential need for alternative strategies in challenging anatomical scenarios where standard approaches are impractical.

## Introduction

A CRT-D device combines cardiac resynchronization therapy (CRT) with an implantable cardioverter-defibrillator (ICD), designed to both resynchronize left ventricular contractions in patients with heart failure and deliver defibrillation shocks in response to arrhythmias. It is indicated for patients with systolic dysfunction, typically with a reduced ejection fraction, as seen in this patient, to prevent sudden cardiac death and improve ventricular function [1].

Subclavian vein obstruction, whether due to stenosis or complete occlusion, poses a significant challenge in the transvenous implantation of CRT-D leads, which typically require an unobstructed path through this vein for proper placement. This complication can result from prior catheterizations, central venous devices, thrombosis, or anatomical variants, severely complicating venous access [2].

The preferred technique for CRT-D implantation involves transvenous lead placement via the subclavian vein. The presence of vascular anomalies like subclavian vein obstruction significantly complicates CRT-D implantation, a generally standardized procedure. Such anomalies force the operator to modify the approach, potentially increasing the technical complexity, procedural duration, and risk of lead malposition. This is clinically significant because venous obstruction not only increases the risk of procedural failure but also necessitates alternative venous access routes [3].

The combination of an obstructed subclavian vein on one side and an excessively acute angle of the subclavian vein on the other side is extremely rare. An incidentally found subclavian vein obstruction is exceedingly rare and has been found at a rate of 0.6% (n = 154) in some studies [4-5]. This unique anatomical configuration can significantly complicate medical procedures, particularly the placement of a cardioverter-defibrillator (CRT-D).

Subclavian vein obstruction prevents access on one side, while an acute angle on the other side hampers proper lead placement, underscoring the need for meticulous pre-procedural planning and alternative strategies to ensure successful outcomes in similar complex cases.

This case report highlights the significance of such a scenario, where the standard approach for implantable cardioverter-defibrillator (ICD) implantation was rendered impractical due to these unusual venous anomalies.

## Case presentation

Clinical presentation

A 69-year-old female presented to the hospital for the scheduled implantation of a cardiac resynchronization therapy defibrillator (CRT-D). Two months prior, she had experienced a myocardial infarction, leading to a coronary angiography and subsequent stent placement. The patient reported a history of heart failure with a left bundle branch block, for which CRT-D implantation was recommended.

Physical examination

On admission, the patient had a blood pressure of 132/75 mmHg, which was slightly elevated. Her heart rate was 61 beats per minute with sinus rhythm, and her temperature was 36.5°C. Respiratory rate was 18 breaths per minute, with an oxygen saturation of 96%.

The patient was oriented to time and place. Pulmonary examination revealed vesicular breath sounds without added sounds, and cardiological examination showed no murmurs or thrills. Abdominal examination was unremarkable, and no peripheral edema was noted.

Clinical history

The patient’s medical history includes a myocardial infarction with subsequent coronary angiography and stent placement, performed two months prior to her current presentation. She has ischemic cardiomyopathy, complicated by mitral and aortic valve insufficiency. Her heart failure is classified as NYHA Class II. She has a history of Grade III arterial hypertension per ESC/ESH guidelines, with a recorded peak of 200/110 mmHg. A left bundle branch block was also noted on the prior ECG.

During the course of treatment, the patient was administered captopril 25 mg, spironolactone 25 mg, amiodarone 200 mg once per day, heparin 5000U (5 ml), amoxicillin trihydrate and potassium clavulanate (865 mg/125 mg) twice per day, cefazolin 1 g, diazepam 5 mg, metamizole 50% (2 ml), pantoprazole 40 mg, potassium chloride 4% (200 ml), and NaCl 0.9% (500 ml). Additionally, supportive oxygen was provided.

Laboratory studies

Primary lab studies showed a slightly increased lymphocyte percentage of 36% (normal 25-33%). The complete blood count was otherwise normal. The coagulogram showed a slightly elevated INR of 1.25 (normal 0.8 to 1.1), while other coagulation measures were normal. Creatinine was normal at 97 µmol/L (normal 53-97.2 µmol/L) (Table 1).

**Table 1:** Laboratory studies conducted prior to attempted CRT-D implantation CRT-D: cardioverter-defibrillator

Parameters		Laboratory values	Reference range
Complete blood count (CBC)	Hemoglobin / HGB	13.4g/dL	12.0-16.0g/dL
	Erythrocytes / RBC	4.7 million/mm^3^	3.5-5.5 million/mm^3^
	Leukocytes / WBC	7,100/mm^3^	4500-11,000/mm^3^
	Thrombocytes/ PLT	207,000/mm^3^	150,000-400,000/mm^3^
	Neutrophil band cells	3%	3-5%
	Segmented neutrophils	53%	35-65%
	Eosinophils	2%	1-3%
	Lymphocytes	36%	25-33%
	Mean corpuscular hemoglobin (MCH)	27.6 pg/cell	25-35 pg/cell
	Mean corpuscular volume (MCV)	80 μm^3^	80-100 μm^3^
Coagulogram	Prothrombin time (PT)	14 seconds	11-15 seconds
	International normalized ratio (INR)	1.25	0.8-1.1
	Fibrinogen	2.8 g/L	2.0 to 4.0 g/L

Electrocardiogram (ECG) findings demonstrated a left bundle branch block with sinus rhythm, a QRS duration of 170 ms, and a heart rate of 61 bpm.

Echocardiographic findings

Echocardiography indicated signs of cardiomyopathy, mitral, and aortic valve insufficiency. A dilated left atrium (5.6cm), and a dilated left ventricle with increased end-diastolic (6.4cm) and end-systolic diameters (5.7cm). The ejection fraction was very low at 18% with asynchronous contraction, anterior septal wall akinesia, and hypokinesia of other walls. The right ventricle was also dilated with an end-diastolic diameter of 2cm, and the tricuspid valve was found to be fibrotic (2.8cm). Mitral regurgitation (2+) and aortic regurgitation (2.5+) were noted, with maximal pulmonary pressure of 30mmHg. The patient presented a very high risk of sudden cardiac death and was recommended for ICD implantation.

CRT-D implantation procedure

**Figure 1:**
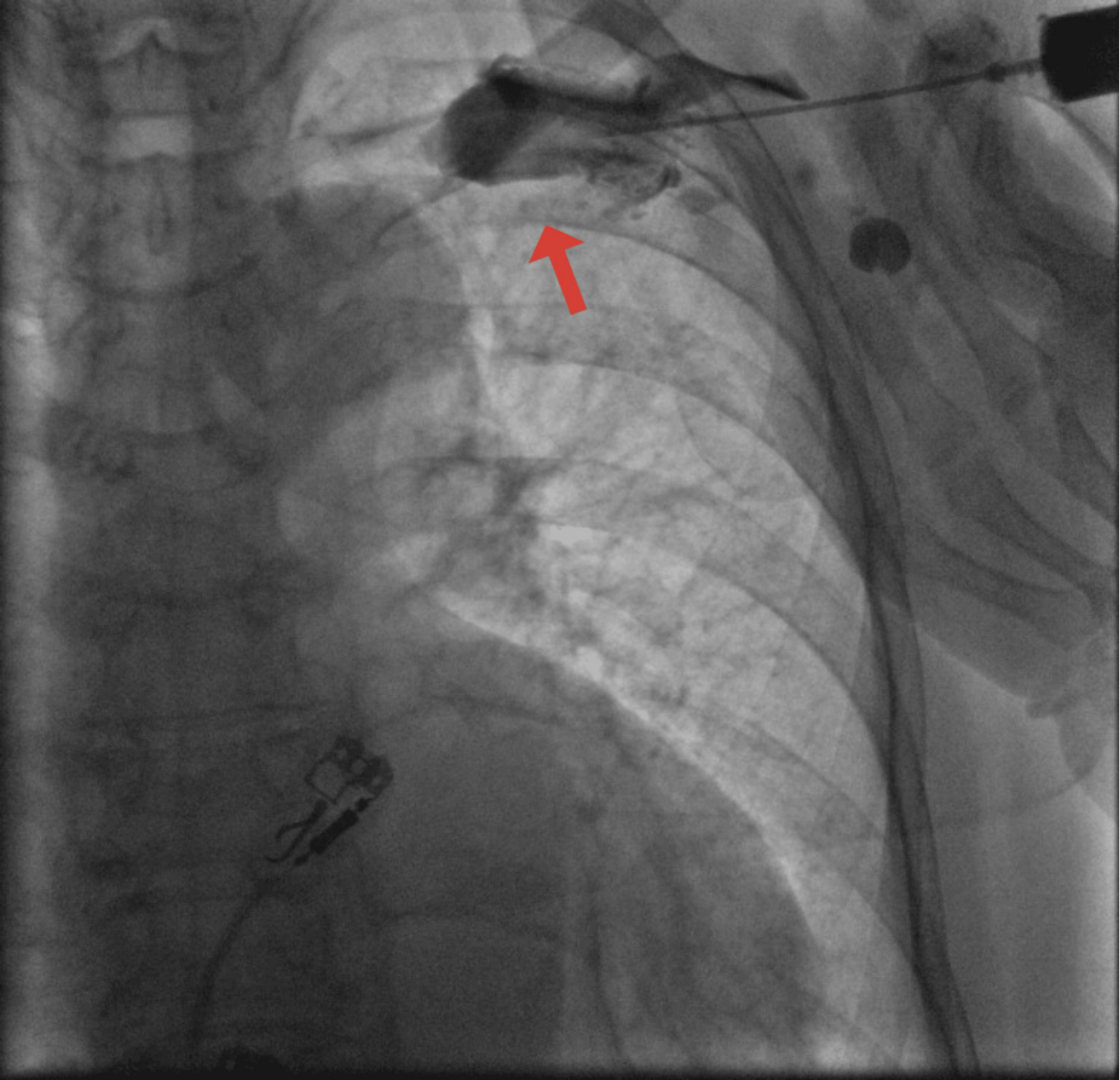
Chest X-ray angiography with contrast shows a thrombosed subclavian vein on the left side.

A skin incision was made in the subclavian vein area. The left subclavian vein was punctured, but the stylet did not pass through. Chest X-ray angiography with contrast showed that the left subclavian vein was dilated and blind-ended at the level of the brachiocephalic artery; additionally, small venous branches could be seen (Figure 1).

**Figure 2:**
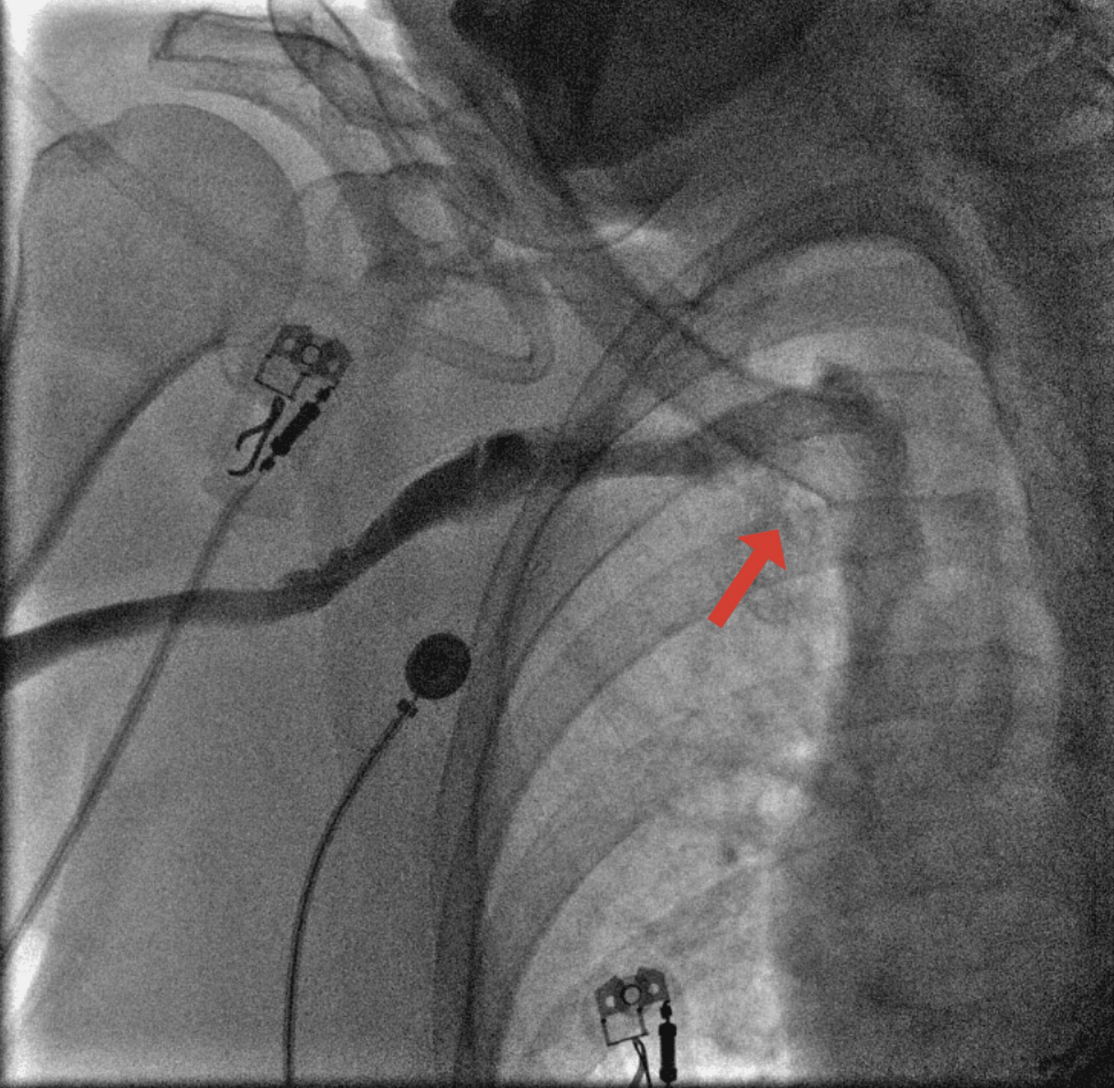
Chest X-ray angiography with contrast shows an acute angle of the subclavian vein on the right side.

The procedure was switched to the right subclavian vein. The right subclavian vein was patent but had two acute angles: subclavian and near the brachiocephalic artery, making the implantation of a cardiac resynchronization therapy defibrillator (CRT-D) impossible (Figure 2).

The procedure was halted due to complications, and CT angiography was recommended to assess the vascular anatomy. Meanwhile, the patient was transferred to the cardiac intensive care unit for monitoring and further evaluation. Thoracotomy was also considered as a backup if implantation proved challenging under CT guidance. However, the patient opted to defer both the CT-guided implantation and thoracotomy, as the procedure was initially scheduled as a preventative measure to reduce the risk of sudden cardiac death and was not urgently required due to the absence of a life-threatening acute heart condition. In the interim, anticoagulation therapy and heart failure management were continued.

## Discussion

In general, a subclavian vein thrombosis in relation to CRT-D implantation is a complication of the procedure; however, incidental discovery of this condition is rare in the literature, especially when combined with an exceedingly acute angle on the other side making the procedure impossible. To further highlight the rarity of the case presented, we conducted a comprehensive literature review of relevant and comparable cases, as well as an analysis of available statistics pertaining to such anatomical anomalies and associated thrombosis.

In a study involving 23 patients, an acute angle was noted in 40% of the cases, and 12% of them exhibited subclavian thrombosis. Successful venipuncture and subsequent cannulation of the subclavian vein were achieved with the first or second passage of the needle in 22 patients, representing 85% of the 26 patients studied [6].

Six patients, accounting for 1.3%, experienced venous occlusion, with five involving the left subclavian vein and one the left innominate vein. Notably, there was a significantly higher incidence of previous cancer in the occlusion group compared to the non-occlusion group (50% vs. 11.5%, p = 0.03) [7]. Bilateral subclavian vein obstruction has also been documented in a patient with Synovitis-acne-pustulosis-hyperostosis-osteitis syndrome (SAPHO syndrome) [8].

CRT-D implantation is associated with its own set of complications; in one study, 9.5% of patients experienced complications such as lead-related re-interventions, local infections requiring re-intervention, CIED-related systemic infections or endocarditis, pneumothorax requiring drainage, cardiac perforation, etc. Of these, lead-related re-interventions were the most common [9]. Additionally, a study following pacemaker patients undergoing hemodialysis found that 80% developed symptomatic subclavian vein obstruction [10]. In one study, a complication rate of 12.5% was observed with respect to the subclavian vein approach for CRT-D implantation, of which 96.3% were successful [11].

The studies referenced highlight that obstructions of this nature are most often related to the procedure itself and are rarely observed pre-procedurally. In our case, the discovery of bilateral obstruction, with one side presenting an acute angle, underscores the exceptional rarity of this case.

The primary treatment for subclavian vein obstruction remains anticoagulation therapy and the use of thrombolytics [12]. Some studies indicate that balloon angioplasty has been used successfully to treat occluded subclavian veins [13]. In cases of occlusion due to thrombosis, thrombolytics successfully obliterated thrombi in 84% of patients [14]. 

As for other methods of CRT-D implantation when the subclavian procedure is not feasible, various case reports and articles offer alternatives. In one such case, a minimally invasive approach using mini-thoracotomy was employed for CRT-D reimplantation after complications arose from skin necrosis due to prior burn injuries. The leads were placed surgically: right atrial and right ventricular leads were introduced through the right atrial appendage, while the left ventricular lead was inserted transapically. This novel combination of transatrial and transapical lead placement serves as an alternative method when conventional transvenous approaches are not feasible [15]. Another case report has described using minimal thoracotomy and endocardial dual chamber lead placement as an alternative CRT-D implantation method when faced with subclavian vein obstruction [3].

Other studies have shown subclavian vein obstruction following CRT-D device implantation [16-17]. However, finding an asymptomatic obstruction incidentally during the insertion of a CRT-D device remains exceedingly rare.

## Conclusions

The unexpected finding of a thrombosed left subclavian vein, combined with an acute angulation of the right subclavian vein, presented significant challenges for CRT-D implantation. Combined incidental discovery of such vascular anomalies is exceedingly rare and underscores the need for alternative access strategies during procedures. Comprehensive preoperative evaluations are essential to identify potential anatomical barriers, allowing for adaptations in approach. Enhanced imaging techniques such as ultrasound-guided insertions, procedural planning and readiness for alternative methods of insertion such as mini-thoracotomy may further improve management of these rare complications, ultimately benefiting patients undergoing complex cardiac device implantations. This case emphasizes the necessity for ongoing research into alternatives for patients unable to undergo CRT-D implantation through the subclavian vein to better understand the implications of vascular anomalies on cardiac interventions.
